# Updating Systematic Reviews: An International Survey

**DOI:** 10.1371/journal.pone.0009914

**Published:** 2010-04-01

**Authors:** Chantelle Garritty, Alexander Tsertsvadze, Andrea C. Tricco, Margaret Sampson, David Moher

**Affiliations:** 1 The Ottawa Methods Centre, Clinical Epidemiology Program, Ottawa Hospital Research Institute, Ottawa, Canada; 2 Department of Public Health Sciences, Faculty of Medicine, University of Toronto, Toronto, Canada; 3 Department of Information Studies, University of Wales, Aberystwyth, United Kingdom; 4 Children's Hospital of Eastern Ontario Research Institute, Ottawa, Canada; 5 Department of Epidemiology and Community Medicine, Faculty of Medicine, University of Ottawa, Ottawa, Canada; Johns Hopkins Bloomberg School of Public Health, United States of America

## Abstract

**Background:**

Systematic reviews (SRs) should be up to date to maintain their importance in informing healthcare policy and practice. However, little guidance is available about when and how to update SRs. Moreover, the updating policies and practices of organizations that commission or produce SRs are unclear.

**Methodology/Principal Findings:**

The objective was to describe the updating practices and policies of agencies that sponsor or conduct SRs. An Internet-based survey was administered to a purposive non-random sample of 195 healthcare organizations within the international SR community. Survey results were analyzed using descriptive statistics. The completed response rate was 58% (n = 114) from across 26 countries with 70% (75/107) of participants identified as producers of SRs. Among responders, 79% (84/107) characterized the importance of updating as high or very-high and 57% (60/106) of organizations reported to have a formal policy for updating. However, only 29% (35/106) of organizations made reference to a written policy document. Several groups (62/105; 59%) reported updating practices as irregular, and over half (53/103) of organizational respondents estimated that more than 50% of their respective SRs were likely out of date. Authors of the original SR (42/106; 40%) were most often deemed responsible for ensuring SRs were current. Barriers to updating included resource constraints, reviewer motivation, lack of academic credit, and limited publishing formats. Most respondents (70/100; 70%) indicated that they supported centralization of updating efforts across institutions or agencies. Furthermore, 84% (83/99) of respondents indicated they favoured the development of a central registry of SRs, analogous to efforts within the clinical trials community.

**Conclusions/Significance:**

Most organizations that sponsor and/or carry out SRs consider updating important. Despite this recognition, updating practices are not regular, and many organizations lack a formal written policy for updating SRs. This research marks the first baseline data available on updating from an organizational perspective.

## Introduction

Systematic reviews (SRs) have become a gold standard for evidence-based decision-making, and are the key building blocks for clinical practice guidelines (CPGs), and health technology assessments (HTAs). Since evidence is continually evolving, results from SRs are prone to change over time and, if ignored, can undermine their validity.[1] To maximize the potential of evidence from SRs, it is important to continually consider currency of information and to emphasize the relevance of keeping them up to date.

Few of the estimated 2500 new English language SRs indexed annually in Medline are reported as updates[2] according to a proposed definition for updating.[3] It is suggested that indicators for updating may occur frequently, and within relatively short timelines.[1] Signals for updating can be quantitative (i.e., a change in statistical significance using a conventional threshold or a change in the magnitude of effect estimate) or qualitative in nature (i.e., including ‘a different description of effectiveness, a new harm that would alter decision-making, a better alternate therapy, a caution that affects clinical decision-making, or the growth of treatment to a new patient group.’). Nonetheless, the level at which updates are undertaken remains unclear as does the basis upon which updating is conducted (e.g., ad hoc versus a formal process).

Healthcare organizations are both large-scale consumers and producers of evidence from SRs. Some organizations have made recommendations about the frequency by which the evidence base needs to be updated in order to keep it up-to-date and valid.[4], [5] For example, the Cochrane Collaboration recommends that SRs be assessed for the need of updating every two years, or a commentary be provided to explain why this was done less frequently.[4] As with the conduct of original SRs, Cochrane's commitment to updating is predicated on authors volunteering of their time to periodically ensure a review is current. This is thought to be unique from that of other organizations that are more likely prompted to update based on immediate need, and which is usually accompanied by specific funding.

Emerging research suggests that updating SRs according to fixed time intervals is perhaps too basic an approach for what appears to be a complex issue influenced by several factors, such as the context in which ‘update’ decisions are made, approaches to monitoring the literature, how meaningful changes or signals are defined in relation to SRs that when detected may trigger updating, and subsequent update procedures used.[6] Nonetheless, generally little is known about the frequency of updating policies and practices across organizations.

It has not yet been determined how best to balance being up-to-date with the resources required to achieve this goal. The lack of adequately developed globally coordinated, reasonable and cost-effective methodologies for updating may be why those who conduct and/or fund SRs do not commonly update. On several levels it seems advantageous to work towards development of the most efficient updating approaches, which must start with an understanding of current updating experiences. Therefore, the aim of this survey was to examine and describe the updating policies and procedures used by healthcare organizations that produce and/or sponsor SRs worldwide.

## Methods

The survey was developed with input from a team of methodologists and systematic reviewers, and was guided by a conceptual framework on updating SRs.[6] The survey consisted of 48 questions (including skip-logic functionality) on the following topics: a) updating policies; b) responsibility for updating; c) changes in estimates of outdated reviews; d) updating strategies and practices (e.g., including when to update, how to update, surveillance, and triggers impacting updating decisions; e) barriers and facilitators to the updating process; f) views on harmonization of updating; the openness to collaboration between groups; and g) descriptive characteristics of the organization and the representative key informant. For the purposes of this research, an ‘update’ was defined as ‘*a discrete event aiming to search for and identify new evidence to incorporate into a previously completed SR; with new evidence taken to mean any evidence not included in the previously completed SR irrespective of its chronological appearance in the literature*.’[3] In addition, the term harmonization was defined as *‘the coming together of different organizations that are involved in the funding, conduct, or reporting of SRs in order to bring into line or to harmonize on issues of conduct, reporting and policy as it relates to updating SRs.’* In order to ascertain what organizations view ‘updating policy’ to mean (i.e., mere guidance or a set of formal procedures implemented either informally or on a compulsory basis etc.) no formal definition was provided to respondents. All items provided non-response options (e.g., not sure) and participants were allowed to skip questions they did not wish to answer. A pilot was administered (January 29^th^ to April 4^th^, 2007) to a sub-sample of 22 organizations including the Evidence-based Practice Centers as designated by the Agency for Healthcare Research and Quality (AHRQ).[7]

The survey was provided to participants via the Survey Monkey web-based service;[8] a suitable format given distribution of the sample across a wide international geographical area and that key informants had email addresses and therefore were likely familiar with Internet use.[9], [10] Emails were sent directly to organizational Directors or to the highest ranking scientific or administrative official asking them to identify the most appropriate internal respondent to answer the survey, which took an estimated 20 to 30 minutes to complete.

Recommended survey methods were employed to maximize Internet survey participation including offering a small fiscal incentive to all participants (e.g., a $10 gift certificate from Amazon.com or iTunes).[10]–[13] The main survey was administered between April 12^th^ and June 8^th^, 2007 with reminder emails sent at approximately 1, 3 and 6 weeks from the first point of contact.

Using a purposive non-random sampling approach, traditional organizations or groups commonly involved in undertaking and/or funding SRs were sampled as potential respondents. The sample was expanded to include entities involved in HTAs and CPGs to allow for stratification by type of evidence synthesis conducted. Key international membership lists of established networks and associations known in the field (i.e., Health Technology Assessment International (HTAi), International Association of Health Technology Assessment (INAHTA), and Guidelines International Network (G-I-N)) were used to create the sampling pool of relevant research organizations. In addition, the 52 Cochrane Collaboration Review Groups (CRGs) were invited to participate in the survey. Thus, a direct attempt was made to broadly select organizations likely to provide the most relevant knowledge and insight into updating. The final sampling frame consisted of 195 different organizations.

Closed-ended questions were analyzed using a descriptive summary of findings in the form of frequencies and percentages. In addition, other details reported in the text were summarized in tabular form. A subgroup analysis was performed comparing CRGs to non-Cochrane organizations in terms of their responses across select updating characteristics.

Participating organizations are not identified in the results as only aggregate data are reported. The institutional ethics review boards of the University of Toronto and the Children's Hospital of Eastern Ontario approved the survey.

## Results

**Figure 1:**
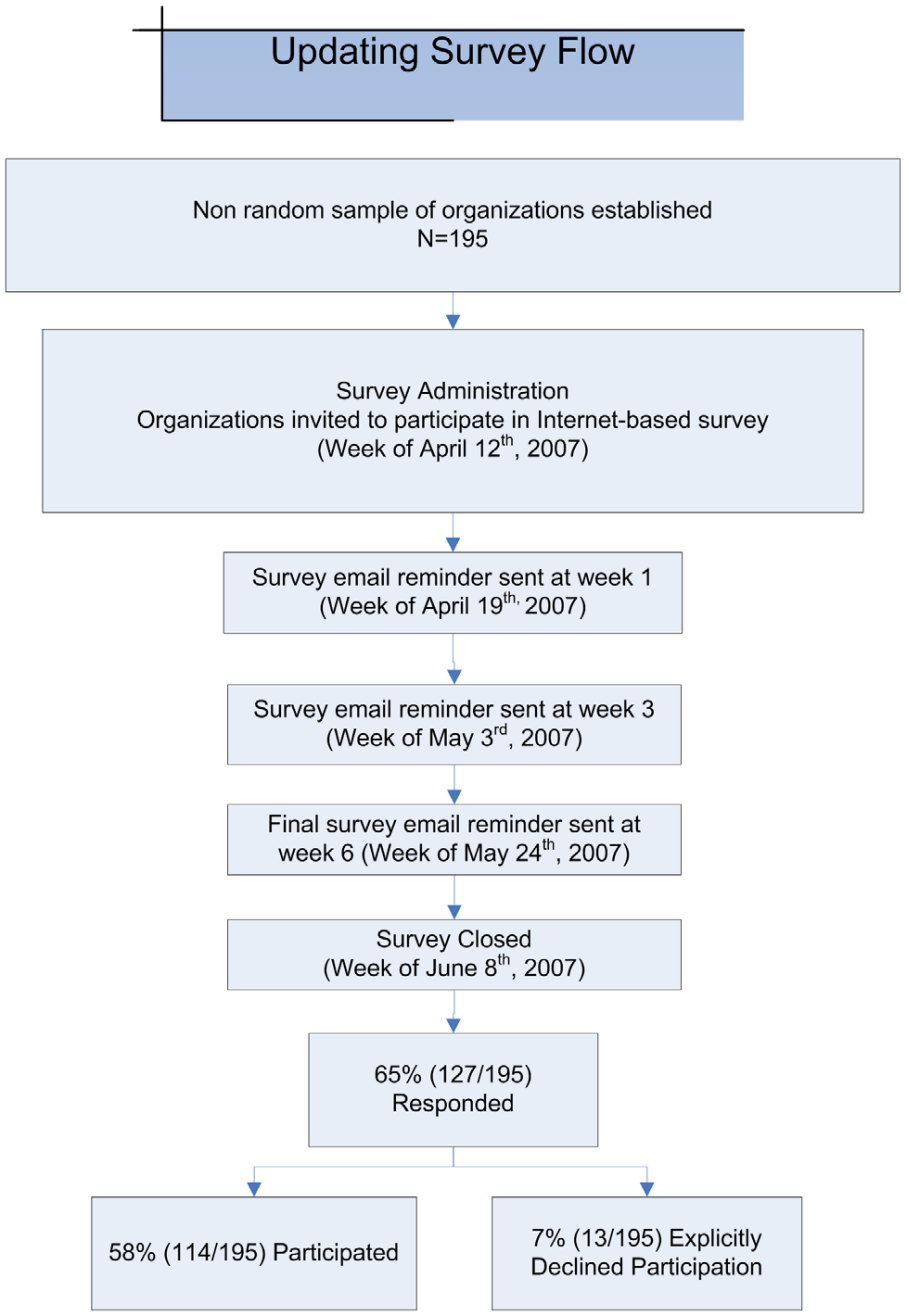
Updating Survey Flow Diagram.

Of the 195 Internet surveys sent by email, 127 organizations responded yielding an overall response rate of 65%. Of those organizations that initially responded, 10% (13/127) formally declined participation and 58% (114/195) completed the survey. Eighty-eight percent (100/114) of respondents completed more than 80% of all questions. (Figure 1. Survey Flow Diagram)

### Characteristics of the Respondent Organizations

Organization respondents (n = 114) were from 26 countries with the United Kingdom, the United States, Canada and Australia accounting for 62% of the sample. (Table 1)

**Table 1:** Organization Responses by Country.

Country	N of Respondents; %
United Kingdom	35/114; 30.7%
United States	15/114; 13.1%
Canada	11/114; 9.6%
Australia	10/114; 8.7%
Netherlands	5/114; 4.4%
Spain	4/114; 3.5%
Brazil	3/114; 2.6%
Denmark	3/114; 2.6%
Finland	3/114; 2.6%
Germany	3/114; 2.6%
Italy	3/114; 2.6%
New Zealand	3/114; 2.6%
Switzerland	3/114; 2.6%
Argentina	1/114; 0.9%
Austria	1/114; 0.9%
Belgium	1/114; 0.9%
France	1/114; 0.9%
Hong Kong	1/114; 0.9%
Latvia	1/114; 0.9%
Malaysia	1/114; 0.9%
Mexico	1/114; 0.9%
Norway	1/114; 0.9%
Poland	1/114; 0.9%
Portugal	1/114; 0.9%
Romania	1/114; 0.9%
Taiwan	1/114; 0.9%

*Percentages rounded to 1st decimal point.

The majority of organizations identified themselves as producers of SRs (75/107; 70%). (Table 2) Of groups that responded, 96% (96/100) indicated they were mainly not-for-profit agencies, academic institutions (40/100; 40%) or national government agencies (21/100; 21%). Government research or infrastructure grants accounted for the majority of funding (85/100; 85%). Yearly organizational expenditures reported for updating ranged widely with the largest proportion of organizations (26/85; 31%) spending $10,000 or less (U.S.) on this activity. (Table 2)

**Table 2:** Organization General Demographics.

Characteristics of Respondent Organizations:		N of Respondents; %
**Type of SR involvement:**	Producers of SRs	75/107; 70%
	Funders of SRs	5/107; 5%
	Both Funders & Producers	27/107; 25%
**Primary Funding Structure:**	Not-for profit	96/100; 96%
	For profit	4/100; 4%
**Category of Organizations:**	Academic Institution	40/100; 40%
	National Government Agency	21/100; 21%
	Regional/Local Government Agency	7/100; 7%
	Private Organization	4/100; 4%
	Medical Specialty Society	4/100; 4%
	Managed Care Organization	0/100; 0%
	Disease Specific Society	0/100; 0%
	Other	24/100; 0%
**Funding:**	Industry/Private Sector	16/100; 16%
	Government Grants	85/100; 85%
	Non-profit (academic; non-governmental)	49/100; 49%
	Endowment fund	4/100; 4%
	Internal Institutional Funds	17/100; 17%
	Other	9/100; 9%
**Annual Expenditures on Updating**	≤$10,000 USD	26/85; 31%
	$11,000–40,000 USD	12/85; 14%
	$41,000–100,000 USD	12/85; 14%
	$100,000–$200,000 USD	6/85; 7%
	$200,000–$500,000 USD	4/85; 5%
	>$500,000 USD	0/85; 0%
**% of Research Expended Annually on Updating**	≤10%	32/97; 33%
	11–40%	42/97; 43%
	41–70%	7/97; 7%
	71–100%	5/97; 5%
	Not sure	4/97; 4%
	Not applicable	7/97; 7%

*For several of the above characteristics, participants were asked to “check all that apply” thus certain totals/percentages do not add up to 100. Percentages rounded to the nearest whole number.

**Figure 2:**
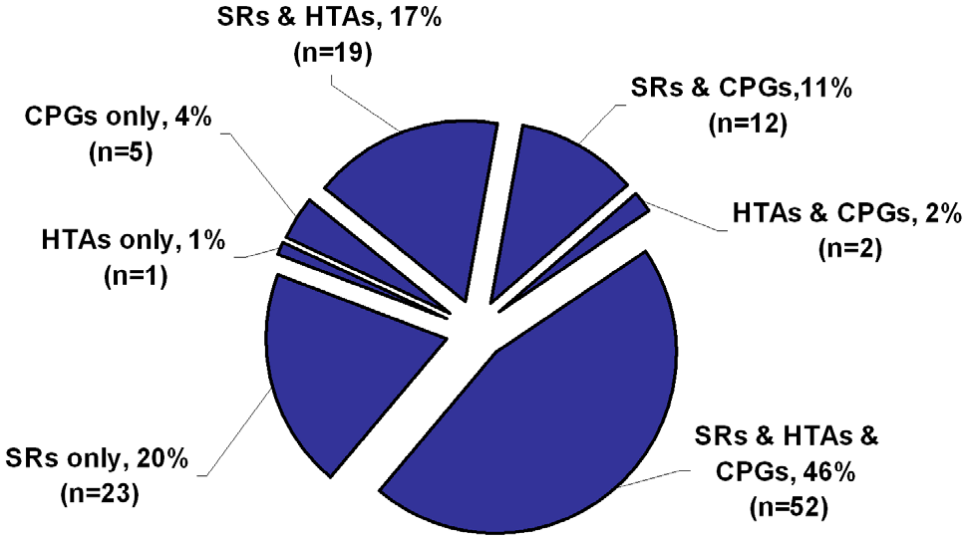
Organizational Involvement in Evidence Synthesis (n = 114).

Twenty percent of all respondent organizations (23/114) reported taking part solely in SRs while 46% percent of organizations (52/114) reported wider concurrent participation in evidence synthesis including SRs, HTAs and CPGs. (Figure 2) Overall, 65% (34/52) of Cochrane Collaboration Review Groups (CRGs) responded thus accounted for 30% (34/114) of all respondents.

### Characteristics of Participant Respondents

Individual respondents identified themselves as co-authors of SRs (70/100; 70%); project manager/coordinator of SRs (61/100; 61%) or as a lead author of a review (59/100; 59%).(Table 3) Further, respondents indicated having received specific training in SRs vis-à-vis workshops (69/100; 69%); participation in lectures (50/100; 50%), university-level training or research (46/100; 46%), or continuing education courses (41/100; 41%).

**Table 3:** Respondent General Demographics.

Characteristics of Participant Respondents:		N of Respondents; %
**Respondent Experience with SRs:**	Lead Author	59/100; 59%
	Co-author	70/100; 70%
	Information Specialist	8/100; 8%
	Statistician	3/100; 3%
	Methodologist/Epidemiologist	41/100; 41%
	Clinical Expert	15/100; 15%
	Editor	45/100; 45%
	Project Manager/Coordinator	61/100; 61%
	None	3/100; 3%
	Other Research Capacity	11/100; 11%
**Respondent Training in SRs:**	University-level training	46/100; 46%
	Continuing Education course(s)	41/100; 41%
	Workshop training	69/100; 69%
	Lecture(s)	50/100; 50%
	No formal training	8/100; 8%
	Other	12/100; 12%

*For several of the above characteristics, participants were asked to “check all that apply” thus certain totals/percentages do not add up to 100. Percentages rounded to the nearest whole number.

### General Findings

Approximately 96% of the survey sample (103/107) ‘strongly’ or ‘somewhat’ agreed with our definition of updating.[3] The majority of respondents (84/107; 79%) viewed the importance of updating SRs as high or very high.

Of the respondent organizations, 57% (60/106) indicated having an updating policy. However, when asked to elaborate on their policies, only 29% (35/106) of them made reference to a written policy; the most common of which was the Cochrane Collaboration's (29/35; 83%). Of the organizations with no formal update processes, 52% (24/46) indicated establishing a formal policy was something their organization would view as important.

#### Updating Practices

A minority of respondents (35/105; 33%) reported regular updating, whereas 59% (62/105) reported updating practices as irregular. In addition, 8% (8/105) of the organizations reported not to engage in any updating practices. Over half of the organizations (53/103; 51%) judged over 50% of their SRs as out of date.

#### Responsibility

Respondents thought that authors of the original review (42/106; 40%), the funder(s) of the original review (16/106; 15%), and policy-makers utilizing the evidence (14/106; 13%) were most accountable for updating SRs. We note that 16% of respondents suggested that responsibility for updating was a collective effort including all the above mentioned with the additional responsibility of information specialists involved with SRs while information specialists separately were thought of as least responsible (2/106; 2%).

#### Surveillance

Regarding updating methods and strategies used to monitor the literature, 63% (66/105) of respondents claimed to be engaged in regular literature searches to identify new evidence while 28% (29/105) reported no such activity. Of groups reporting to search regularly, search frequencies varied: 11% (7/65) searched monthly; 9% (6/65) searched every six months; 20% (13/65) searched annually; and 11% (7/65) searched every 18 months. Forty-five percent (29/65) indicated ‘other’ search time intervals that ranged from every 3 to 36 months.

When monitoring the literature in an attempt to identify new evidence, the two most frequently reported monitoring strategies (sometimes, often, or always) were: conducting general literature searches including electronic and hand searches (81/102; 79%), and contacting experts in the field (70/99; 71%). Additional surveillance strategies included surveillance of SRs (66/101; 65%), HTAs and CPGs (65/101; 64%), automatic alerts or use of surveillance software (59/98; 59%), monitoring trial registries (49/100; 49%), and statistical approaches (11/98; 11%).

Of organizations reporting to monitor the literature, 74% (46/62) reported to ‘always’ or ‘often’ use the same search strategy of the original SR while 43% (25/48) reported to use a *modified* search. Eighteen percent (9/50) reported ‘always’ or ‘often’ developing a *new* search strategy therefore discarding the original.

#### Influences

Stakeholders that reportedly influence an organization's decision-making process of funding or conducting SR updates were: authors of the original SR (either within or external to the organization) (75/101; 74%), external policy-makers (72/100; 72%); the organization administration as the funder of the original SR (65/100; 65%) and experts in the field (62/100; 62%). Information specialists (39/97; 40%) and statisticians (11/97; 11%) were least likely to impact this decision. Over one-third (34/99; 35%) of organizations indicated that patients or consumer groups ‘sometimes’ influence this decision-making process.

When assessing specific issues that factor into determining ‘when’ to update, a formal request from a policy or healthcare decision-maker was the most frequently cited factor by the majority of respondents (80/99; 81%) followed by number of new studies identified (77/100; 77%. Additional issues are provided in Table 4.

**Table 4:** Factors that Impact on Determining ‘When’ to Update.

Factors impacting ‘when’ to update:	N of Respondents; %
Formal request from a policy or healthcare decision maker	80/99; 81%
Number of new studies identified	77/100; 77%
Totality (comprehensiveness) of all new evidence or data including harms & benefits	75/99; 76%
Reporting of serious or ‘new’ serious adverse events	74/100; 74%
Time credibility	69/96; 72%
New inclusion criteria (outcomes; interventions; populations; methodological advances/new analysis)	59/93; 63%
Need for an internal organizational decision	52/92; 57%
Number of participants in new studies	44/96; 46%

*Percentages rounded to the nearest whole number.

#### Conducting Updates

Most organizations are seldom utilizing current existing methods, for example cumulative meta-analytic approaches, when undertaking updating. The most frequently used approach is a pre-set time based updating frequency (66/99; 67%). (Table 5)

**Table 5:** Update Methods/Procedures.

Update methods/procedures:	Use (always, often or sometimes)/N of respondents; %	Use (seldom or never)/N of respondents; %
Time specific approach	66/99; 67%*	26/99; 26%
Bibliometric database entry-date searching	37/98; 38%	46/98; 47%
Editorial strategy with an algorithm of actions	25/99; 25%	61/99; 62%
Cumulative meta-analysis (or extensions)	11/99; 11%	68/99; 69%
Barrowman's identifying the ‘null’ diagnostic test	4/99; 4%	77/99; 78%

*Percentages rounded to the nearest whole number.

#### Updating Action

**Figure 3:**
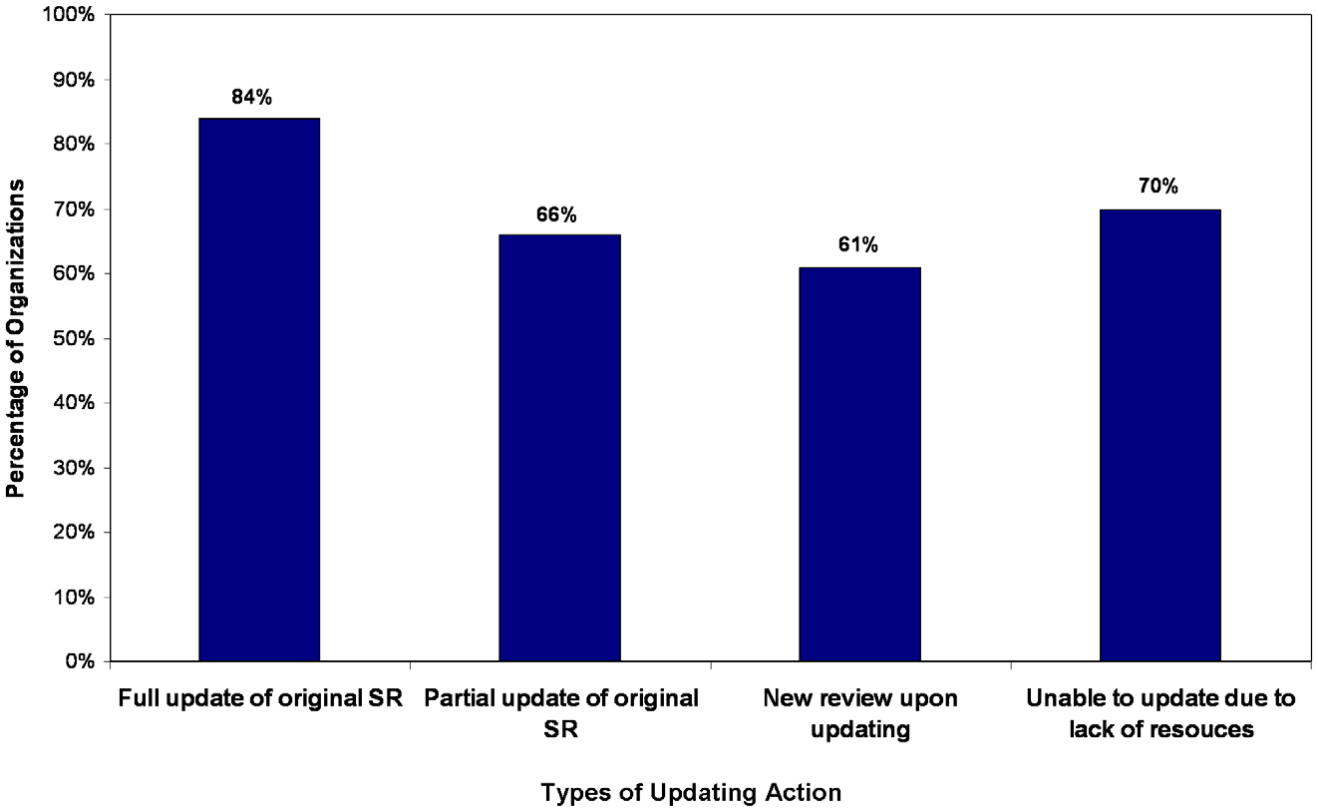
Types of Updating Action (n = 101).

When examining levels of updating, 84% (85/101) of organizations reported (always, often, or sometimes) carrying out full updates of all sections of the original SR; 66% (66/100) reported taking part in partial updates relating to only certain sections of original SRs; and 61% (59/99; 60%) reported participation in conducting an entirely new review upon updating. Seventy percent of groups (71/101) indicated knowing an SR is out of date but were unable to commence updating due to lack of resources (e.g., funding, personnel, or time). (Figure 3)

Seventy-three percent (74/101) of organizations reported ‘sometimes’ or ‘always’ identifying literature published after the date of the last search but before completion of the original SR. Organizations reported that this information is usually incorporated as an addendum in the review (47/101; 47%), or as a formal revision to the analysis (40/101; 40%). Twenty-two percent (22/101) characterized this issue as the following: referred to studies as awaiting assessment, ongoing or unclassified; referenced studies in the section of the review where included; or noted and/or incorporated studies qualitatively into the discussion. An additional 9% (9/101) reported to be unsure as to how their organization dealt with this issue of identifying literature post-hoc.

Forecasting the need for a future update within the text of a SR was reported by 53% (52/99) groups. Withdrawal of at least one SR from circulation after judging the review as out of date was reported by 56% (55/98) of groups, while formal retirement of at least one SR when deemed out of date, or no longer in need of investigation was noted by 38% (37/97) of respondents.

Approximately, 54% of organizations (56/103) reported the ability to (always or often) draw on the same people involved in the original SR.

#### Barriers

Respondent organizations reported several moderate to serious barriers when updating. These included the perceived need to do the following: redo data extraction (44/97; 45%); re-assess study quality (38/95; 38%); change the original screening criteria (31/97; 32%) and change the original search strategy (25/97; 26%). Other broad barriers identified (moderate to serious) included: limited funding and resources (72/100; 72%); reviewer motivation (53/96; 55%); limited academic credit for updating work (49/100; 49%); limited publishing formats (35/100; 35%) and having to update reviews done by others (35/99; 35%).

#### Harmonization

A large portion of respondents (70/100; 70%) indicated they ‘somewhat’ or ‘strongly’ support centralizing updating efforts across institutions or agencies that produce SRs (i.e., harmonizing updating efforts). The most common perceived benefits (moderate to major) of participating in international harmonization efforts for updating were the use of existing resources more efficiently (79/101; 78%) and access to new information, ideas, materials or other resources (79/101; 78%). (Table 6)

**Table 6:** Major/Moderate Benefits to Harmonization of Updating.

Benefits to harmonization:	N of respondents; %
Use of existing resources more efficiently	79/101; 78%
Access to new information, ideas, materials or other resources	79/101; 78%
Potential to minimize duplication of services	77/101; 77%
Ability to address issues beyond a single organization's domain	62/100; 62%
Share responsibility across organizations for complex/controversial issues	54/99; 55%

*Percentages rounded to the nearest whole number.

**Figure 4:**
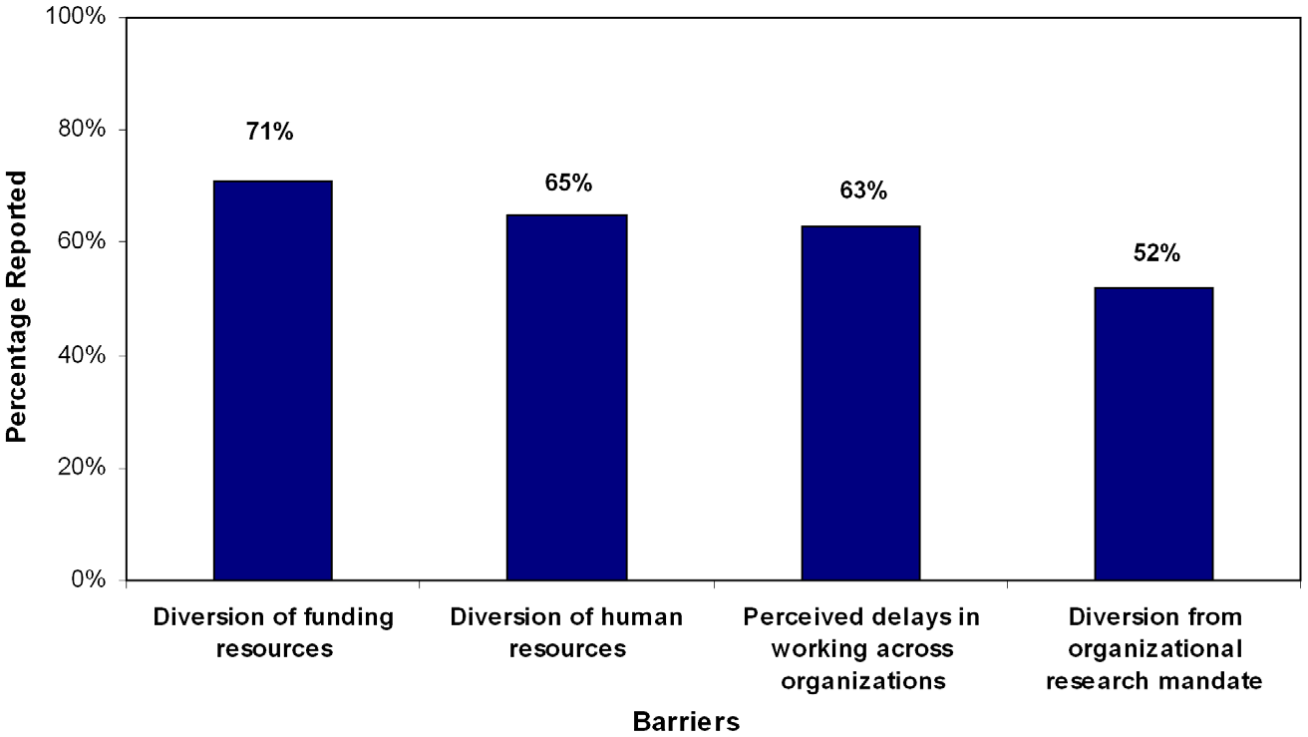
Major/Moderate Barriers to International Harmonization of Updating Efforts (n = 97).

Respondents also indicated several barriers to harmonization including the possible diversion of organizations' funding resources (69/97; 71%) and insufficient human resources (63/97; 65%). As well, 63% of responders (61/97) viewed perceived delays in working across organizations, and possibly diverting the focus of research mandates within organizations (52/97; 52%) as moderate to serious barriers to collaboration. (Figure 4)

Obstacles aside, 84% (83/99) of the respondents indicated they ‘somewhat’ to ‘strongly’ favour the development of a central registry of SRs, which would be similar to efforts within the clinical trials community.

#### Sensitivity Analysis by Cochrane Review Groups (CRGs)

Statistically significant differences were noted between Cochrane Review Groups (CRGs) and non-CRG organizations (non-CRGs) across certain updating characteristics. (Table 7) For example, 31.8% (CI −48.9%, −11.40%) fewer CRGs (38.2%) described their general updating practices as irregular compared to non-CRGs (70%); 59.6% (CI 39.1%, 72.1%) more CRGs (86.2%) viewed authors as most responsible for updating compared to non-CRGs (26.6%); 25% (CI −38.6%, −10.3%) fewer CRGs (0%) perceived funders as responsible for updating versus non-CRGs (25%); and 16.9% (CI −28.6%, −1%) fewer CRGs (3.4%) identified policy-makers as bearing responsibility compared to non-CRGs (20.3%).

**Table 7:** Sensitivity Analysis of Cochrane Review Groups (CRGs) versus Other Organizations.

Variables	CRGs % (n)	Non-CRGs % (n)	Absolute Differences (%)	95% Confidence Intervals	
**Agreement with the Definition of Update**	94.3% (33/35)	97.2% (70/72)	−2.9%	−16%,	5.0%
**Views the Importance of Updating as ‘High’ to ‘Very High’**	97.1% (34/35)	69.4% (50/72)	27.7%	−12.7%,	39.3%
**Has a Policy on Updating SRs**	97.1% (33/34)	37.5% (27/72)	59.6%	−42.9%,	70.1%
**General Organization Updating Practice**					
Updates Regularly	61.8% (21/34)	20.0% (14/70)	41.8%	−21.9%,	58.0%
Updates Irregular	38.2% (13/34)	70.0% (49/70)	−31.8%	−48.9%,	−11.4%
Does Not Update	0% (0/34)	10.0% (7/70)	−10.0%	−19.2%,	1.4%
**Perceived as Most Responsible for Updating**					
Funders	0% (0/29)	25.0% (16/64)	−25.0%	−36.8%,	−10.3%
Authors	86.2% (25/29)	26.6% (17/64)	59.6%	39.1%,	72.1%
Information Specialists	0% (0/29)	3.1% (2/64)	−3.1%	−10.7%,	8.8%
Policy-makers	3.4% (1/29)	20.3% (13/64)	−16.9%	−28.6%,	−1.0%
All Combined	10.3% (3/29)	25.0% (16/64)	−14.7%	−28.3%,	3.7%
**Conducts Regular Literature Searches to Survey the Literature (Yes)**	84.8% (28/33)	61.3% (38/62)	23.5%	4.3%,	38.6%
**Estimated Frequency of Literature Searching for Updating**					
Monthly searching	6.7% (1/15)	27.8% (5/18)	−21.1%	−44.9%,	6.6%
Every 6 mos searching	26.7% (4/15)	22.2% (4/18)	4.5%	−23.4%,	33.0%
Every 12 mos searching	26.7% (4/15)	44.4% (8/18)	−17.7%	−44.7%,	14.4%
Every 18 mos searching	40.0% (6/15)	5.6% (1/18)	34.4%	5.9%,	59.1%
**Search Strategies Typically Used (Always, Often, Sometimes) When Monitoring Literature**					
Same Search Found in the Original SR	96.0% (24/25)	83.3% (30/36)	12.7%	−5.2%,	28.2%
Modified Search	100% (23/23)	97.0% (32/33)	3.0%	−11.5%,	15.3%
New Search	43.8% (7/16)	45.2 (14/31)	−1.4%	−28.2%,	26.7%
**Sources Used (Always, Often, Sometimes) to Monitor the Literature During Surveillance**					
General Literature Searches	100% (32/32)	74.2% (49/66)	25.8%	11.8%,	37.4%
Automatic Database Alerts	60.0% (18/30)	66.7% (40/60)	−6.7%	−27.3%,	13.2%
Monitoring Trials Registries	77.8% (21/27)	45.2% (28/62)	32.6%	10.4%,	49.1%
Statistical Approaches	3.7% (1/27)	17.2% (10/58)	−13.5%	−25.6%,	2.9%
Monitoring Systematic Reviews	64.3% (18/28)	75.0% (48/64)	−10.7%	−31.2%,	8.4%
Monitoring CPGs and/or HTAs	60.0% (18/30)	74.6% (47/63)	−14.6%	−34.5%,	4.9%
Contacting Experts in the Field	65.6% (21/32)	77.8% (49/63)	−12.2%	−31.4%,	6.1%
**Inability to Update Outdated SRs Due to Lack of Resources**	81.3% (26/32)	72.6% (45/62)	8.7%	−10.4%,	24.3%
**Ability to Draw on the Same Reviewers for Updating**	100% (31/31)	86.4% (57/66)	13.6%	0.9%,	23.9%
**Procedures Used to Determine ‘When to Update’ a SR**					
Time-specific	96.8% (30/31)	59.0% (36/61)	37.8%	20.5%,	50.6%
Editorial Strategy	39.3% (11/28)	24.1% (14/58)	15.2%	−4.9%,	35.6%
Entry Date Searching	33.3% (8/24)	49.2% (29/59)	−15.9%	−35.6%,	7.6%
Cumulative Meta-analyses (CMA)	4.0% (1/25)	18.5% (10/54)	−14.5%	−27.3%,	3.0%
**Perceived Barriers (Serious to Moderate) to Updating SRs Completed by Others**					
Changes Required to the Original Search Strategy	30.4% (7/23)	31.0% (18/58)	−0.6%	−20.2%,	22.3%
Changes Required to the Screening Criteria	50.0% (12/24)	33.3% (19/57)	16.7%	−6%,	38.2%
Need to Re-do Data Extraction	62.5% (15/24)	50.9% (29/57)	11.6%	−11.8%,	32.3%
Need to Re-do Quality Assessment	58.3% (14/24)	42.1 (24/57)	16.2%	−7.2%,	37.2%
**Perceived General Barriers (Serious to Moderate) to Updating**					
Updating Methodologies	10.3% (3/29)	24.6% (16/65)	−14.3%	−27.8%,	4.1%
Funding Resources	67.7% (21/31)	77.3% (51/66)	−9.6%	−29.1%,	8.3%
Redundancy of Updating (Motivation Level of Reviewers)	100% (30/30)	39.0% (23/59)	61.0%	44%,	72.4%
Limited Academic Credit	60.0% (18/30)	48.4% (31/64)	11.6%	−9.8%,	31.0%
Limited Publishing Formats	33.3% (9/27)	42.6% (26/61)	−9.3%	−28.6%,	12.8%
Having to Updating SRs Completed by Others	60.0% (15/25)	33.9% (20/56)	26.1%	1%,	44.3%
**Agreement with Harmonization of Updating Efforts Across Organizations**	66.7% (18/27)	80.0% (52/65)	−13.3%	−33.8%,	5.2%
**Perceived Benefits to Forming International Harmonization Updating Efforts**					
Access to New Information, Ideas, Materials or Other Resources	88.5% (23/26)	83.6% (56/67)	4.9%	−13.9%,	17.9%
Potential to Minimize Duplication of Services	76.9% (20/26)	85.1% (57/67)	−8.2%	−28.2%,	7.8%
Use of Existing Resources More Efficiently	80.8% (21/26)	87.9% (58/66)	−7.1%	−26.7%,	7.6%
Ability to Address Issues Beyond a Single Organization's Domain	64.0% (16/25)	70.8% (46/65)	−6.8%	−28.5%,	13.0%
Shared Responsibility Across Organizations for Complex or Controversial Issues	71.4% (15/21)	60.0% (39/65)	11.4%	−12.6%,	30.5%
**Perceived Barriers to Forming International Harmonization Updating Efforts**					
Diversion of Human Resources	68.0% (17/25)	75.4% (46/61)	−7.4%	−29%,	11.7%
Diversion of Funding Resources	66.7% (16/24)	85.5% (53/62)	−18.8%	−39.9%,	0.0%
Diversion from Organization's Research Mandate	65.2% (15/23)	61.3% (38/62)	3.9%	−19.3%,	24.2%
Perceived Delays in Working Across Organizations	73.9% (17/23)	68.8% (44/64)	5.1%	−17.6%,	23.4%
**Agreement (Strongly or Somewhat) with Development of a Central Registry of SRs**	76.7% (23/30)	89.6% (60/67)	−12.9%	−31.3%,	2.1%

*Percentages rounded to 1st decimal point.

In addition, 23.5% (CI 4.3%, 38.6%) more CRGs (84.8%) conducted regular literature searches to monitor the literature versus non-CRGs (61.3%); 25.8% (CI 11.8%, 37.4%) more CRGs (100%) reported using general literature searches as a key source of new evidence when monitoring the literature in relation to non-CRGs (74.2%); 32.6% (CI 10.4%, 49.1%) more CRGs (77.8%) reported to survey trials registries as a source of new evidence compared to non-CRGs (45.2%); and 34.4% (CI 5.9%, 59.1%) more CRGs (40%) also reported to conduct searches at 18-month intervals versus non-CRGs (5.6%). However, no differences were noted between groups at monthly, six month or 12-month search periods. Again, 13.6% (CI 0.9%, 23.9%) more CRGs (100%) indicated the ability to draw on same reviewers for updating versus non-CRGs (86.4%); 37.8% (CI 20.5%, 50.6%) more CRGs (96.8%) used a time-specific approach to determining the need to update compared to non-CRGs (59.0%); and 61% (CI 44.0%, 72.4%) more CRGs (100%) indicated reviewer motivation level as a barrier to updating versus non-CRGs (39%). The same held true for having to update SRs completed by others whereby 26.1% (CI 1.0%, 44.3%) more CRGs (60%) perceived this as a barrier compared to non-CRGs (33.9%). However, 18.8% (CI 0.0%, −39.9%) fewer CRGs (66.7%) perceived the diversion of funding resources as an obstacle to international harmonization efforts versus non-CRGs (85.5%).

## Discussion

We believe that this is the first survey to examine updating practices of organizations engaged in knowledge synthesis. This survey is guided by a conceptual framework,[6] has a response rate higher than those typically obtained from Internet surveys (58%),[11] and includes strong international representation.

Importantly, this survey revealed inconsistencies between the belief of the importance of updating and limited updating activity among respondents outside the Cochrane Collaboration (nearly 70% of the respondents). Analysis of Cochrane Review Groups (CRGs) and non-Cochrane organizations showed several significant differences in approaches to updating. Most fundamental is the Cochrane Collaboration's policy that Cochrane Reviews should be assessed for updating within two years of publication and its policy that authors must agree to keep reviews up-to-date when registering with the Collaboration.[4] Not surprisingly, the CRGs perform updates in greater numbers than the other entities who responded.

Within the Cochrane Collaboration, responsibility for updating resides predominantly with the authors. This did yield an advantage in that CRGs were able to draw on the same review team for updating to a greater extent than non-Cochrane respondents. Still, when reviewers had responsibility for updating, reviewer motivation was the most prominent barrier to updating – every responding CRG reported this to be a moderate or serious obstacle, compared to only 39% of those who did not place primary responsibility with the original authors.

For both Cochrane and non-Cochrane respondents, funding is the prominent barrier to updating, with 70% reporting inadequate resources. As many of these groups estimated that half or more of their SRs were outdated, supporting updates becomes the crucial issue to address within the overall allocation of evidence synthesis funding. Value of Information (VOI) analysis is a possible mechanism for establishing the costs and benefits of further information gathering and subsequent organizational priority-setting.[14] It may have relevant application to the field of updating SRs. A conceptual updating framework[6] and evidence-based update decision-making tools are needed to guide this process.

Placing the onus for updating mainly on authors of SRs has had some success within the Cochrane Collaboration although may not be a practical approach for agencies that do not share its values and culture. Journal publishers and academic organizations can contribute to overcoming some of the known motivational challenges faced by authors with updating duties.[15] Academic institutions can support updating by according academic recognition on par with conducting and publishing original SRs. Journals can increase publishing outlets for updates, for instance, when accepting a review for publication, by also committing to publishing any future updates. Organizations can make updates more prominent by tying them to the original review. For example, the Public Library of Science (PLoS) electronically links SR updates to freely available original reports.[16] Updates are also indexed in PubMed and Medline provided that authors explicitly identify that the review is an update of a previously published article.[17] Although useful, currently few SRs are cited as updates in Medline.[2]

Despite the paucity of methodological tools for updating,[18] and limited adoption of those that do exist [Table 5], very few organizations reported lack of updating methodologies as barriers to updating. Most reported composite factors as the drivers for when to update, and most reported using a variety of established methods to identify new evidence. Thus, the problem seems largely a lack of resources – financial and human. Therefore solutions that either infuse additional resources or greatly reduce the work required for updating seem most needed.

One approach may be for organizations to cooperate in harmonizing updating efforts, sharing resources and knowledge on issues of surveillance, conduct, reporting and policy for updating. The idea of harmonizing efforts was supported by the majority of organizations responding to this survey but such cooperation is in its infancy.

Although the generalizability of the survey findings is limited due to the use of a non-random sampling frame, it is apparent that, at the time of the survey, several organizations lacked a formal policy for maintaining the SRs that they produce. Given that it has been empirically demonstrated that review findings are overturned by new evidence,[1] often within short time horizons, it is likely prudent for those organizations to address this issue. Even if resources are not available for updating, some mechanism is necessary to monitor the emerging evidence so that SRs, or the guidelines upon which they are based, can be formally withdrawn when they can no longer inform best practice. Agencies, as opposed to individual authors who might undertake a SR purely as a scientific endeavour, have a responsibility to manage the information they sponsor or commission throughout its lifecycle.

Updating is a complex and resource intensive process, often weighed down by barriers, and it needs to be balanced with other research endeavours. Nonetheless, updating should be viewed as a worthy undertaking that ensures health practice and policy are based on the best and most current evidence.
